# Supplementary material for: The zebrafish orthologue of familial Alzheimer’s disease gene PRESENILIN 2 is required for normal adult melanotic skin pigmentation
Source: PLoS One. 2018 Oct 25;13(10):e0206155. doi: 10.1371/journal.pone.0206155 (PMC6201934; doi:10.1371/journal.pone.0206155)
Supplement: S3 Table — (DOCX) [file pone.0206155.s007.docx]

**S3 Table. *In situ* hybridization against *tbx16* transcripts in DoLA neurons.**

| DoLA numbers in +/+ | DoLA numbers in *N140fs*/+ | DoLA numbers in *N140fs*/*N140fs* |
| --- | --- | --- |
| 18 | 18 | 25 |
| 24 | 20 | 21 |
| 24 | 25 | 23 |
| 25 | 19 | 26 |
| 24 | 20 | 25 |
| 21 | 21 | 21 |
| 17 | 28 | 21 |
| 19 | 26 | 22 |
| 24 | 24 | 23 |
| 17 | 20 | 20 |
|  | 17 |  |
|  | 17 |  |
|  | 17 |  |
|  | 26 |  |
|  | 25 |  |
|  | 24 |  |
|  | 21 |  |
|  | 25 |  |
|  | 20 |  |
|  | 23 |  |
|  | 25 |  |
|  | 18 |  |
|  | 16 |  |
|  | 26 |  |
