# Supplementary material for: The zebrafish orthologue of familial Alzheimer’s disease gene PRESENILIN 2 is required for normal adult melanotic skin pigmentation
Source: PLoS One. 2018 Oct 25;13(10):e0206155. doi: 10.1371/journal.pone.0206155 (PMC6201934; doi:10.1371/journal.pone.0206155)
Supplement: S4 Table — Copies per 25ng (assuming complete reverse transcription of total RNA). (DOCX) [file pone.0206155.s008.docx]

**S4 Table.** **Allele-specific expression analysis of *N140fs/+* embryos (non-treated and CHX–treated) at 50 hpf in 25ng of total embryo cDNA.** Copies per 25ng (assuming complete reverse transcription of total RNA).

| *psen2* wild type allele | |
| --- | --- |
| Non-treated *N140fs*/+ | CHX-treated *N140fs*/+ |
| 194.99 | 462.66 |
|  | |
| *N140fs* mutant allele | |
| Non-treated *N140fs*/+ | CHX-treated *N140fs*/+ |
| 43.63 | 244.37 |
