# Supplementary material for: The zebrafish orthologue of familial Alzheimer’s disease gene PRESENILIN 2 is required for normal adult melanotic skin pigmentation
Source: PLoS One. 2018 Oct 25;13(10):e0206155. doi: 10.1371/journal.pone.0206155 (PMC6201934; doi:10.1371/journal.pone.0206155)
Supplement: S1 File — (DOCX) [file pone.0206155.s009.docx]

**S1 File. Mutation screening and breeding.**

In addition to the mutation *N141I*, another missense mutation in the human *PSEN2* gene, *V148I*, was also considered for introduction into zebrafish *psen2*. *V148I* is a missense mutation caused by a G-to-A transition at the first position of codon 148 of *PSEN2* [[1](#_ENREF_1)]. Like *N141I*, this mutation is also located within TMD2. However, since both valine and isoleucine are hydrophobic (with the hydrophobicity of isoleucine slightly stronger than that of valine), the PolyPhen-2 prediction score [[2](#_ENREF_2)] for *V148I* (0.222 with sensitivity of 0.91 and specificity of 0.88) is more benign than that for *N141I* (i.e. 0.934). The age of onset of Alzheimer’s disease in the individual with the *V148I* mutation was age 71 [[1](#_ENREF_1)], which is also much later than the mean onset age for the *N141I* mutation (age 53.7). It is also reported that *V148I* does not significantly change the Aβ 42/40 ratio [[3](#_ENREF_3)].

**Mutation screening and breeding of the CRISPR/Cas9 and template oligo-injected G0 fish**

The *in vivo* cleavage activity of the CRISPR/Cas9 system used in this study was tested using the T7 endonuclease I assay. It is interesting that even the uninjected TU embryos showed two cleavage bands in a T7 endonuclease I assay, although the sizes of these bands were different to those expected for the samples injected with the CRISPR/Cas9 system (S1A Fig). This may due to that the T7 endonuclease I assay system is so sensitive that it cleavage can occur at sites of single mismatched bases heteroduplexes [[4](#_ENREF_4)], i.e. due to single nucleotide polymorphisms (SNPs). The CRISPR/Cas9 system-injected TU embryos showed four cleavage bands in the T7 endonuclease I assay (S1A Fig), two of which were the same as those for the uninjected TU embryos (possibly caused by SNPs in the TU genome), while the other two were the sizes expected for mutation produced by the DNA cleavage activity of the CRISPR/Cas9 system. Therefore, the CRISPR/Cas9 system used in this study showed cleavage activity in the TU genome, and we could use this system to attempt to generate the mutations desired by co-injecting it together with homology-directed repair (HDR) oligonucleotide templates.

**S1 Fig. T7 endonuclease assays and mutation-specific PCRs for embryos at 24 hpf.**

(A) T7 endonuclease I assay for testing the cleavage activity of the CRISPR/Cas9 system. (B) “*N140I*” allele-detection PCR for testing of CRISPR/Cas9 plus “N140I oligo” co-injected TU embryos. 10 embryos from each injection batch were pooled for these tests. Both batches of the injected TU embryos showed positive signals in the “N140I” allele-detection PCR. Therefore, some of these “N140I oligo” injected TU embryos may have carried the “N140I” allele in the genomes of some cells. (C) “*V147I*” allele-detection PCR for testing the CRISPR/Cas9 plus “V147I oligo” injected TU embryos. 10 embryos from each batch were pooled for these tests. Both batches of the injected TU embryos showed positive signals from the “*V147I*” allele-detection PCR. Therefore, some of these “V147I oligo” injected TU embryos may have carried the “*V147I*” allele in the genomes of some cells. (D) T7 endonuclease I assay for detecting random mutations at the CRISPR/Cas9 target site in the F1 progeny. Tail-clip biopsies from 46 of the F1 progeny from the CRISPR/Cas9 plus “V147I oligo” injected mosaic G0 fish were tested using the T7 endonuclease I assay to screen for the presence of cells with mutations at the target site. Only 5 fish showed cleavage patterns indicating the presence of mutations.

For the “N140I oligo” injected TU embryos, 10 embryos from each batch were pooled and tested using the “*N140I*” allele-specific detection PCR (S1B Fig). Positive signals showed that there should be mutant fish carrying the “*N140I*” mutation in both batches. The remaining G0 embryos were raised to adulthood and then genotyped using the “*N140I*” allele-specific detection PCR (S2A Fig). Twelve of these genotyped G0 fish (from a total of 120) showed positive signals in the “*N140I*” allele-specific detection PCR (S2A Fig). All these “*N140I*” allele-carrying G0 fish were then outbred with TU wild type fish to generate F1 fish potentially heterozygous for the “*N140I*” allele. 10 F1 embryos from each “*N140I*” allele carrying G0 fish crossed with a TU wild type fish were pooled for the “*N140I*” allele-specific detection PCR. Unfortunately, none of these progeny showed any positive signal from the “*N140I*” allele-specific detection PCR, indicating that the “*N140I*” allele was not present in the F1 progeny. Interestingly, the “*N140I*” allele-specific detection PCR for the F1 progeny from one “*N140I*” allele-carrying G0 fish produced a DNA fragment of ~400 nucleotides in size (S2B Fig), while the positive signal for the “*N140I*” allele-specific detection PCR was calculated to be ~300 nucleotides. This probably indicates the existence of a DNA rearrangement at the cleavage site that incorporated all or part of the oligonucleotide sequence in an unintended manner.

**S2 Fig. Mutation-specific PCR tests of G0 and F1 fish.**

(A) “*N140I*” allele-specific detection PCRs on tail-clip biopsies from G0 fish. Twelve G0 fish (120 in total) showed positive signals in the “N140I” allele-specific detection PCR. (B) “*N140I*” allele-specific detection PCRs from F1 embryos of the G0 mosaic fish showing “*N140I*” allele-positive signals. 10 F1 embryos at 24 hpf from each “*N140I*” allele-carrying G0 fish were pooled for testing. The F1 progeny from one “*N140I*” allele-carrying G0 fish showed a signal at ~400 bp, which may result from imperfect incorporation of the “N140I oligo” sequence into the target site of the CRISPR/Cas9 system. (C) “*V147I*” allele-specific PCRs from F1 embryos of the G0 mosaic fish showing “*V147I*” allele-positive signals. 10 F1 embryos at 24 hpf from each “*V147I*” allele carrying G0 fish were pooled for testing. The F1 progeny from one of the “*V147I*” allele-carrying G0 fish showed the same positive signal as the injected G0 embryos. (D) “*V147I*” allele-specific detection PCR from tail-clip biopsies of F1 fish. Two out of twelve tested F1 fish (raised from the positive batch of embryos observed above in D) showed positive signals, indicating they might carry the desired “*V147I*” allele. (E) “*V147I*” allele-specific detection PCR using the same forward primer as in (E) but a different reverse primer binding farther downstream in *psen2* DNA. While the pooled F1 embryos still gave a positive signal, the two F1 fish no longer showed a positive signal using this PCR, revealing that the previously seen positive signals (in E) were artefacts.

For the “V147I oligo” injected TU embryos, positive signals were also detected using the “*V147I*” allele-specific detection PCR (S1C Fig, signal from10 embryos pooled). Six of the 36 tested G0 fish showed positive signals using the “*V147I*” allele-specific detection PCR. The six “*V147I*” allele-carrying G0 fish were then outbred with TU wild type fish. 10 embryos from F1 progeny from each “*V147I*” allele-carrying G0 fish with a TU wild type fish were pooled for the “*V147I*” allele-specific detection PCR. The F1 progeny from one of the “*V147I*” allele carrying G0 fish showed the same positive signal as the injected G0 embryos (S2C Fig). It seemed that these F1 progeny might carry the “*V147I*” allele. When these F1 progeny were raised, they were genotyped using the “*V147I*” allele-specific detection PCR. Two out of twelve tested F1 fish showed positive signals, indicating they might carry the “*V147I*” allele. To verify these results, a region encompassing the intended mutation site in these two fish was amplified by PCR using the primers for the T7 endonuclease assay, and then sequenced. However, only wild type DNA sequences were seen. To check subsequently the previous positive “*V147I*” allele-specific detection PCR, the PCR was modified to use the same forward primer (specific to the desired mutation) but a different reverse primer (farther downstream of the previously used reverse primer). Interestingly, although the genomic DNA from the pooled F1 embryos still showed a positive signal, the DNAs from the two F1 positive fish no longer showed a positive signal using this PCR test (S2E Fig). Thus, the previous positive signals detected for these two fish were aretefacts. It seems that, in the pooled F1 embryos, the “*V147I*” allele did exist, but the allele was either at too low a frequency to be likely to be detected using the number of fish that we observed or, possibly, cannot be passed through the germline (i.e. for some reason it may be lethal to gamete formation or embryo development).

In conclusion, in this study we used the CRISPR/Cas9 system to attempt to insert fAD mutations (point mutations) into the zebrafish genome to investigate the pathological changes caused by these mutations. However, while we could detect the likely existence of these point mutations in the G0 fish, we did not see them transmitted to F1 progeny.

**NHEJ-generated mutations at the CRISPR/Cas9 target site in F1 progeny**

46 of the F1 progeny from the “*V147I*” allele-carrying mosaic G0 fish were tested using T7 endonuclease I assays to detect mutations generated through NHEJ. The mutation-carrying F1 progeny showed different cleavage patterns to the wild type fish (S1D Fig), but similar patterns to that from embryos injected only with the CRISPR/Cas9 system without mutation-containing oligonucleotides (S1A Fig), indicating these mutations were likely induced by the injected CRISPR/Cas9 system alone. Among the 46 F1 fish tested, only five fish showed cleavage patterns indicating the presence of mutations. Sequencing across the target site in these five fish showed the presence of only two different mutations. One mutation (carried by two of the fish) was a 15 nucleotide-indel mutation (an in-frame mutation) that deleted two codons (T141 and L142) with an insertion of another seven codons (MISLISV) and was consequently named *psen2^T141_L142delinsMISLISV^* (*T141_L142delinsMISLISV*, Fig 1A). The other mutation (carried by three of the fish) was a seven nucleotide-deletion resulting in a frameshift mutation downstream from N140 leading to a premature termination codon at the 142^th^ codon position. It was consequently named *psen2^N140fs^* (*N140fs*, Fig 1A).

**References**

1. Lao JI, Beyer K, Fernandez-Novoa L, Cacabelos R (1998) A novel mutation in the predicted TM2 domain of the presenilin 2 gene in a Spanish patient with late-onset Alzheimer's disease. Neurogenetics 1: 293-296.

2. Adzhubei IA, Schmidt S, Peshkin L, Ramensky VE, Gerasimova A, et al. A method and server for predicting damaging missense mutations: Nat Methods. 2010 Apr;7(4):248-9. doi: 10.1038/nmeth0410-248.

3. Walker ES, Martinez M, Brunkan AL, Goate A (2005) Presenilin 2 familial Alzheimer's disease mutations result in partial loss of function and dramatic changes in Aβ 42/40 ratios. Journal of Neurochemistry 92: 294-301.

4. Babon JJ, McKenzie M, Cotton RGH (2003) The use of resolvases T4 endonuclease VII and T7 endonuclease I in mutation detection. Molecular Biotechnology 23: 73-81.
