# Supplementary material for: The zebrafish orthologue of familial Alzheimer’s disease gene PRESENILIN 2 is required for normal adult melanotic skin pigmentation
Source: PLoS One. 2018 Oct 25;13(10):e0206155. doi: 10.1371/journal.pone.0206155 (PMC6201934; doi:10.1371/journal.pone.0206155)
Supplement: S2 File — (DOCX) [file pone.0206155.s010.docx]

**S2 File. dqPCR results for allele-specific transcript quantification in six month old brains.**

**S1 Table. Allele-specific transcript quantification in six month old *T141_L142delinsMISLISV*/+ and wild type sibling brains.** Copies per 25ng of total brain cDNA (assuming complete reverse transcription of total brain RNA).

| *psen2* wild type allele | |
| --- | --- |
| +/+ wild type allele under normoxia | *T141_L142delinsMISLISV*/+ wild type allele under normoxia |
| 895.88 | 450.69 |
| 954.56 | 579.27 |
| 827.13 | 309.93 |
| 1196.3 | 531.43 |
| +/+ wild type allele under hypoxia | *T141_L142delinsMISLISV* /+ wild type allele under hypoxia |
| 1187.5 | 250.22 |
| 1048.5 | 561.6 |
| 981.92 | 557.32 |
| 956.9 | 709.39 |
|  | |
| *T141_L142delinsMISLISV* mutant allele | |
| +/+ mutant allele under normoxia | *T141_L142delinsMISLISV*/+ mutant allele under normoxia |
| 2.862 | 565.52 |
| 0.103 | 634.11 |
| 1.2 | 297.33 |
| 2.963 | 604.62 |
| +/+ mutant allele under hypoxia | *T141_L142delinsMISLISV*/+ mutant allele under hypoxia |
| 4.137 | 264.77 |
| 3.156 | 671.13 |
| 2.232 | 656.45 |
| 2.972 | 722.62 |

**S2 Table. Allele-specific transcript quantification in six month old *N140fs*/+ fish and wild type sibling brains.** Copies per 25ng of total brain cDNA (assuming complete reverse transcription of total brain RNA).

| *psen2* wild type allele | |
| --- | --- |
| +/+ wild type allele under normoxia | *N140fs*/+ wild type allele under normoxia |
| 1346.8 | 918.15 |
| 1806.9 | 885.32 |
| 1729.6 | 842.01 |
| 1630.9 | 837.78 |
| +/+ wild type allele under hypoxia | *N140fs*/+ wild type allele under hypoxia |
| 1678.6 | 838.53 |
| 1601.3 | 903.36 |
| 1635.7 | 815.97 |
| 1718.4 | 866.78 |
|  | |
| *N140fs* mutant allele | |
| +/+ mutant allele under normoxia | *N140fs*/+ mutant allele under normoxia |
| 11.017 | 154.64 |
| 29.408 | 165.58 |
| 18.746 | 141.68 |
| 10.673 | 148.65 |
| +/+ mutant allele under hypoxia | *N140fs*/+ mutant allele under hypoxia |
| 7.767 | 164.15 |
| 12.353 | 167.54 |
| 22.487 | 174.94 |
| 10.041 | 161.02 |
