# Supplementary material for: The zebrafish orthologue of familial Alzheimer’s disease gene PRESENILIN 2 is required for normal adult melanotic skin pigmentation
Source: PLoS One. 2018 Oct 25;13(10):e0206155. doi: 10.1371/journal.pone.0206155 (PMC6201934; doi:10.1371/journal.pone.0206155)
Supplement: S3 File — (DOCX) [file pone.0206155.s011.docx]

**S3 File. *In situ* transcript hybridization analysis of DoLA neuron number.**

Blockage of the expression of *psen2* using a morpholino has been shown to increase the number of a particular spinal cord interneuron – the Dorsal Longitudinal Ascending (DoLA) neuron [[1](#_ENREF_1)]. Since the loss of pigmentation phenotype in homozygous *N140fs* mutants is suggestive of loss of γ-secretase activity, we assumed this mutation might result in loss of all *psen2* function. As there is currently no antibody detecting zebrafish Psen2 protein available, we attempted to demonstrate loss of *psen2* function for *N140fs* by observing DoLA neuron number in embryos at 24 hpf.

To examine the effect of *N140fs* on DoLA number, embryos from mating of a pair of heterozygous fish were collected. Theoretically, this family would be comprised of approximately 50% heterozygous mutant, 25% homozygous mutant and 25% wild type genotypes. *In situ* transcript hybridization against transcripts of the gene *tbx16* that labels DoLA neurons [[2](#_ENREF_2)] was then performed on these embryos at 24 hpf. Genotyping of each embryo was performed after the number of DoLA neurons in each embryo had been recorded, using PCRs specific for the mutant and wild type alleles. However, two-tailed t-tests assuming either equal or unequal variances found no significant differences in DoLA number between any two genotypes (S3 Fig), (similar to observations for the *S4Ter* mutation, Jiang, unpublished results), suggesting that genetic compensation may be induced by these endogenous mutations to suppress the phenotype of increased DoLA number after loss of *psen2* activity from morpholino injection [[3](#_ENREF_3)].

**S3 Fig. DoLA neuron numbers.**

DoLA neuron numbers in wild type and *N140fs* mutant embryos as revealed by *in situ* hybridisation against *tbx16* transcripts.

44 embryos from a pair-mating of fish heterozygous for the *N140fs* mutation were subjected to *in situ* transcript hybridisation at 24 hpf. Genotyping subsequent to DoLA quantification showed that this family included 24 heterozygous mutants, 10 homozygous mutants and 10 wild type sibling embryos. Values of p were determined in two-tailed t-tests.

**S3 Table. *In situ* hybridization against *tbx16* transcripts in DoLA neurons.**

| DoLA numbers in +/+ | DoLA numbers in *N140fs*/+ | DoLA numbers in *N140fs*/*N140fs* |
| --- | --- | --- |
| 18 | 18 | 25 |
| 24 | 20 | 21 |
| 24 | 25 | 23 |
| 25 | 19 | 26 |
| 24 | 20 | 25 |
| 21 | 21 | 21 |
| 17 | 28 | 21 |
| 19 | 26 | 22 |
| 24 | 24 | 23 |
| 17 | 20 | 20 |
|  | 17 |  |
|  | 17 |  |
|  | 17 |  |
|  | 26 |  |
|  | 25 |  |
|  | 24 |  |
|  | 21 |  |
|  | 25 |  |
|  | 20 |  |
|  | 23 |  |
|  | 25 |  |
|  | 18 |  |
|  | 16 |  |
|  | 26 |  |

**References**

1. Nornes S, Newman M, Wells S, Verdile G, Martins RN, et al. (2009) Independent and cooperative action of Psen2 with Psen1 in zebrafish embryos. Experimental Cell Research 315: 2791-2801.

2. Tamme R, Wells S, Conran JG, Lardelli M (2002) The identity and distribution of neural cells expressing the mesodermal determinant spadetail. BMC Developmental Biology 2: 9.

3. Rossi A, Kontarakis Z, Gerri C, Nolte H, Hölper S, et al. (2015) Genetic compensation induced by deleterious mutations but not gene knockdowns. Nature 524: 230.
