# Supplementary material for: The zebrafish orthologue of familial Alzheimer’s disease gene PRESENILIN 2 is required for normal adult melanotic skin pigmentation
Source: PLoS One. 2018 Oct 25;13(10):e0206155. doi: 10.1371/journal.pone.0206155 (PMC6201934; doi:10.1371/journal.pone.0206155)
Supplement: S4 File — (DOCX) [file pone.0206155.s012.docx]

**S4 File. Cycloheximide treatment of *N140fs*/+ embryos from 48 to 50 hpf.**

To verify whether NMD is occurring for *N140fs* allele transcripts we treated *N140fs*/+ embryos with cycloheximide at 48 hpf for two hours to stabilize any NMD-targeted mRNA [[1](#_ENREF_1),[2](#_ENREF_2)]. Cycloheximide (CHX) is a potent translation elongation inhibitor, and a short treatment with this drug can be used to stabilize NMD-targeted mRNAs [[1](#_ENREF_1),[2](#_ENREF_2)]. In our study, we treated *N140fs*/+ embryos (generated by pair-mating of a *N140fs*/*N140fs* female and a +/+ male fish) at 48 hpf with 200 μg/mL of cycloheximide (Sigma-Aldrich, St. Louis, Missouri, USA, C4859) at 28.5°C for two hours. After treatment, 20 of the treated *N140fs*/+ embryos were pooled for total RNA extraction with the RNeasy Mini Kit (QIAGEN, Venlo, Netherlands, 74104). 20 non-treated *N140fs*/+ embryos were used as a control group. The RNAs from the pooled embryos, of the treated and non-treated groups, were used to prepare cDNA using the SuperScript™ III First-Strand Synthesis System (Invitrogen, Carlsbad, California, USA, 18080051) and Random Primers (Promega, Madison, Wisconsin, USA, C1181), respectively. A pair of PCR primers (forward primer 5’-AAGAAGACCCGAACTCAGTGG-3’, reverse primer: 5’-CTTGTAGAGCAGCACCAGGATG-3’) were used to amplify a region spanning the mutation site. The PCR cycling conditions were: 95°C, 2 min, and then 31 cycles of [95°C, 30 s; 60°C, 30 s; 72°C, 30 s], 72°C 5 min. The PCR products were predicted to be ~110 nucleotides in length (with fragments amplified from the N140fs deletion mutation allele cDNA being 7 nucleotides shorter than from the wild type allele). The PCR products were separated by electrophoresis through a 20% polyacrylamide gel, at 120V for five hours.

As shown in S4A Fig, PCR on cDNA from the CHX–treated *N140fs/+* embryos, resulted in two bands in the electrophoretic gel of approximately equal intensity, while PCR on cDNA from non-treated *N140fs/+* embryos resulted in only one band at the apparent same size as the upper band of the treated group. However, the bands in S4A Fig are not well resolved due to the small differences in DNA fragment length involved. Therefore, the same digital quantitative PCR (dqPCR) designed for allele-specific expression analysis in *N140fs*/+ brains (see Materials and Methods) was performed on the same cDNA preparations from these the CHX-treated and the untreated embryos. As show in S4B Fig, expression of both the wild type *psen2* allele and the *N140fs* allele was upregulated after CHX-treatment compared to the expression of these alleles in the untreated control. However, the fold change (FC) of the upregulation of the *N140fs* allele (FC=5.601) was significantly higher than that for the wild type *psen2* allele (FC=2.373), supporting that the mutant mRNAs were stabilized by CHX in the *N140fs/+* embryos. This supports that the mutant mRNAs are subject to NMD.

**S4 Fig. dqPCRs detecting wild type and mutant alleles in *N140fs/+* embryos at 50 hpf after two hours of cycloheximide treatment relative to untreated embryos.**

(A) In a 20% polyacrylamide gel, amplification of cDNA fragments spanning the mutation (7 nucleotides shorter than wild type) was only observed in the CHX-treated group, while only one higher molecular weight band (from the wild type allele) was observed in the non-treated group. This supports that NMD destabilizes the mutant transcript in heterozygous embryos. (B) Using dqPCR, both the wild type *psen2* allele and the *N140fs* allele were observed to be increased after the CHX-treatment. The fold change (FC) of the increase of the *N140fs* allele transcripts (FC=5.601) was higher than that for the wild type *psen2* allele transcripts (FC=2.373).

**S4 Table.** **Allele-specific expression analysis of *N140fs/+* embryos (non-treated and CHX–treated) at 50 hpf in 25ng of total embryo cDNA.** Copies per 25ng (assuming complete reverse transcription of total RNA).

| *psen2* wild type allele | |
| --- | --- |
| Non-treated *N140fs*/+ | CHX-treated *N140fs*/+ |
| 194.99 | 462.66 |
|  | |
| *N140fs* mutant allele | |
| Non-treated *N140fs*/+ | CHX-treated *N140fs*/+ |
| 43.63 | 244.37 |

**References**

1. Carter MS, Doskow J, Morris P, Li S, Nhim RP, et al. (1995) A regulatory mechanism that detects premature nonsense codons in T-cell receptor transcripts in vivo is reversed by protein synthesis inhibitors in vitro. J Biol Chem 270: 28995-29003.

2. Hurt JA, Robertson AD, Burge CB (2013) Global analyses of UPF1 binding and function reveal expanded scope of nonsense-mediated mRNA decay. Genome Res 23: 1636-1650.
